# Waterproof, thin, high-performance pressure sensors-hand drawing for underwater wearable applications

**DOI:** 10.1080/14686996.2021.1961100

**Published:** 2021-08-17

**Authors:** Chi Cuong Vu, Jooyong Kim

**Affiliations:** Department of Organic Materials and Fibers Engineering, Soongsil University, Seoul, Republic of Korea

**Keywords:** Waterproof, hand-drawing, flexible sensors, machine learning, underwater applications, 10 Engineering and Structural materials, 208 Sensors and actuators, 212 Surface and interfaces, 306 Thin film / Coatings, Sensors and actuators, surface and interfaces, thin film/coatings

## Abstract

Wearable sensors, especially pressure sensors, have become an indispensable part of life when reflecting human interactions and surroundings. However, the difficulties in technology and production-cost still limit their applicability in the field of human monitoring and healthcare. Herein, we propose a fabrication method with flexible, waterproof, thin, and high-performance circuits – based on hand-drawing for pressure sensors. The shape of the sensor is drawn on the pyralux film without assistance from any designing software and the wet-tissues coated by CNTs act as a sensing layer. Such sensor showed a sensitivity (~0.2 kPa^−1^) while ensuring thinness (~0.26 mm) and flexibility for touch detection or breathing monitoring. More especially, our sensor is waterproof for underwater wearable applications, which is a drawback of conventional paper-based sensors. Its outstanding capability is demonstrated in a real application when detecting touch actions to control a phone, while the sensor is dipped underwater. In addition, by leveraging machine learning technology, these touch actions were processed and classified to achieve highly accurate monitoring (up to 94%). The available materials, easy fabrication techniques, and machine learning algorithms are expected to bring significant contributions to the development of hand-drawing sensors in the future.

## Graphical Abstract

**Figure:**
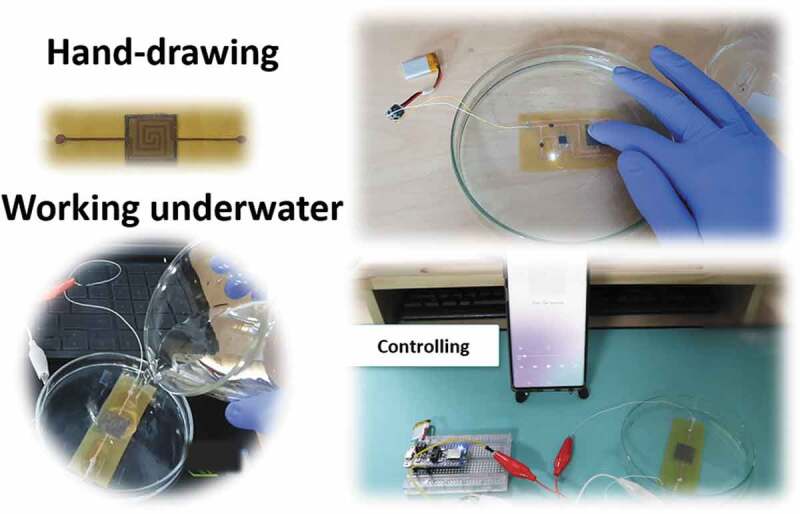

## 1. Introduction

Traditional sensors, typically fabricated from rigid materials, such as metals or semiconductors, are hard, inflexible, and difficult to wear devices. Meanwhile, flexible sensors show superiorities by lightweight, small dimensions, hypoallergenic, and comfortable on the body [1–5].

Flexible pressure sensors, as an aspect of flexible wearable sensors, have attracted a lot of attention from material scientists. Their applications appear in many different research fields, including human motion recognition [6–8], health monitoring of patients [9–11], smart textiles [12–15], or electronic skins [16–20].

There are three main mechanisms to convert force deformations to electronic signals: piezo-resistive, capacitive, or piezoelectric. Among these principles, piezoelectric sensors are made by piezoelectric materials transforming deformation into electrical energy [21–23]. When a mechanical pressure (touch) is applied on certain so-called piezoelectric materials, the electric charges could be separated because of electrical dipole moments, and then an electrical voltage is generated. In this type, sensor elements are self-powered. So, the main advantages of piezoelectric pressure sensors are robustness and low power [24,25]. Meanwhile, the practical applications are restricted due to low flexibility, complexity, and hard-to-integrate into a system.

Capacitive pressure sensors work on the relationship between force and capacitance. Capacitive elements are mechanically simple and robust. So, this sensor-type is able to work over a wide temperature/pressure range. Due to no DC current flows through the capacitor, they are low power, small hysteresis, and suitable for wireless applications. However, the disadvantages of this type are non-linearity and stray capacitance [26–29]. The stray capacitance is additional capacitances caused by connecting cables or components are close together of the circuit. This unwanted capacitance can be confused with signals.

For piezoresistive-type, the sensors include several layers, the most important being two electrode layers separated by a low-conductive thin layer [30–33]. Herein, the resistance changes predominantly as a result of the change in the contact resistance with pressing. Besides, there are some intrinsically piezoresistive materials and sensors, not relying on contact resistance variations. A simple mechanism leads to some advantages in the cost, linear output, high durability, as well as ease of signal collection. However, this type usually suffers from high thickness and poor sensitivity, which limit their applications in e-skins or e-healthcare.

Considering the aspect of easy fabrication, especially sensors based on hand-drawing [34–39], there are some recent studies that showed the ability to create sensors without assistance from design software. For example, Xu et al. [40] reported a variety of e-skin sensors from pencil and paper, including biophysical sensors, sweat biochemical sensors, thermal stimulators, or humidity energy harvesters. Liu et al. [41] also presented a drawing high-performance (0.63 kPa^−1^) capacitive pressure sensors on copying tissues for finger touch detection, motion, and proximity. Costa et al. [42] proposed a pressure sensor system on paper. This hand-drawn system exhibited a linear response for pressures up to 1.2 kPa, and a sensitivity of 51 mV kPa^−1^. However, all of the above studies still have a major limitation on water-resistance. In other words, these sensors are water permeation and impossible to work in environments with high humidity or directly underwater [43]. Besides, there is no good signal processing algorithm for monitoring applications.

To fulfill this aim, we present a hand-drawing pressure sensor using flexible film (pyralux) for electrodes and wet-tissues-coated by carbon nanotubes (CNTs) for the sensing layer. This simple process will help our sensors not only keep the advantages of resistive pressure sensor type (low cost, easy to collect the output signal and integrate into systems) but also extend the sensor limitation in e-skin/healthcare devices by a small thickness, a high-performance at ~0.2 kPa^−1^ for under 6 kPa and ~0.05 kPa^−1^ for over 8 kPa. More significantly, these sensors are waterproof, which is still limited to conventional sensors from cellulose papers. Furthermore, the classification of complex touch actions is achieved by training signal samples with machine learning. The accuracy of classification is 94%. This is a highlight direction for the sensor potential in the human monitoring underwater field.

## 2. Experimental details

### 2.1. Materials

Wet-tissues was acquired from Sangyong C&B Inc., Seoul, South Korea. A flexible film (pyralux) was obtained from DuPont Inc., United States. The characteristics of the pyralux film can ensure good compatibility and flexibility of sensors. Etching powder was prepared from SME Co., Ltd., South Korea. Carbon nanotube inks (CNTs – 0.1 wt%) was obtained from KH Chemicals Co., Ltd, Seoul, South Korea. A double-sided hot melt adhesive film (PU) was prepared for the spacer layer (Sealon Co. Ltd, Seoul, South Korea). And, a thin single-sided tape from 3 M was used to located and protect the area of the electrodes.

**Figure 1.**
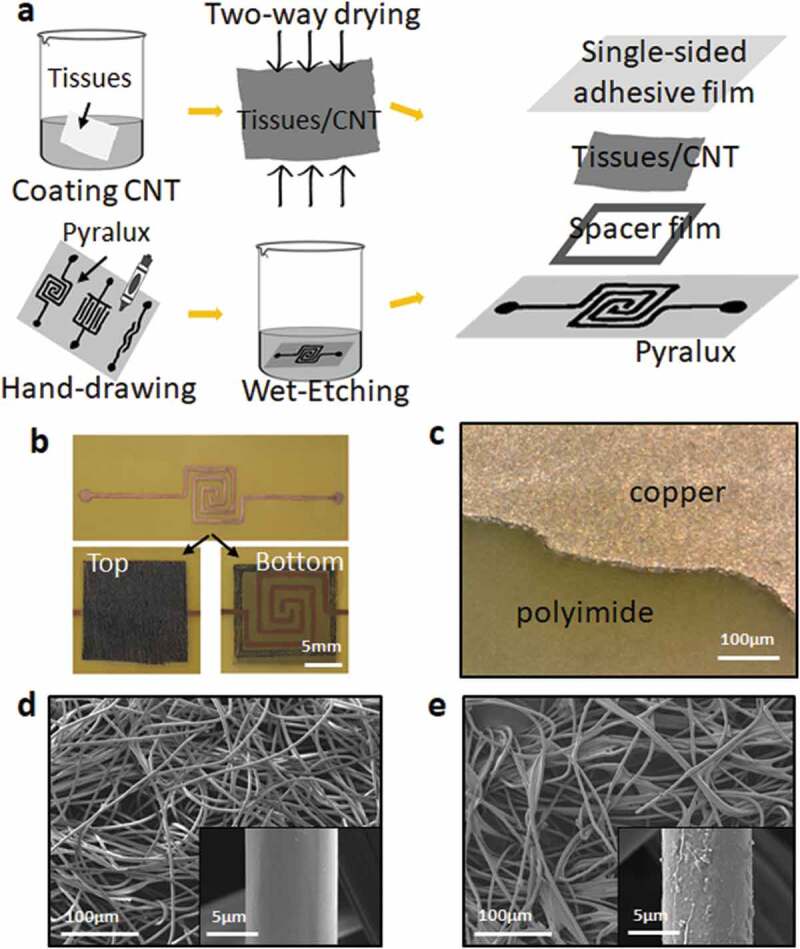
The figure of (a) Sensor fabrication process, (b) Top and bottom side of the sensor, (c) SEM picture of the pyralux film after etching-solution, (d) SEM picture of the sensing layer (wet-tissues) before coated by CNTs, (e) SEM pictures of the sensing layer (wet-tissues) after coated by CNTs

Figure 1(a) shows a schematic illustration of the fabrication process of this pressure sensor. The electrodes can be drawn into arbitrary geometries on a flexible printed circuit board (pyralux film) by a permanent marker pen (electrode distance: 1 mm). One side of it is 1 oz copper, while the other side is polyimide. The material can be manipulated around small areas and will not crack as easily as copper tape. Then, the wet-etching method was used to remove non-electrode parts on the pyralux film for 20 mins. Electrodes were still protected by the marked lines and removed by acetone liquid after etching solution. For the sensing layer, we used wet-tissues-coated by CNT inks and dried (two-way air-drying) at 120°C for 15 mins. CNT ink is composed of CNT powder, solvent, and binder. After dipping, squeezing, heating (drying), the binder will attach the CNT particles to the nonwoven fibers. Besides, the force inter-fibers (friction) also increase the adhesion. However, this adhesive force is not strong, the CNTs will fall out under large deformations, such as many stretching, pressure, bending, twisting cycles, etc., and need a cover layer in actual applications. The sheet resistance of the tissues-CNT is about 30 Ω/sq. Finally, a thin adhesive tape was added to the top side of the sensor to be flexible and waterproof. As shown in Figure 1(b), sensor dimension was found to be 15 × 15 mm. The thickness of the pyralux film is about 0.032 mm. Meanwhile, this value is 0.03 mm for the adhesive tape and 0.16 mm for the tissues/CNTs (Figure S1). Two small electric wires were soldered to each electrode to connect the system. Figure 1(c) shows the surface of pyralux layer after the etching solution at the high magnification.

Scanning electron microscopy images (SEM) of the sensing layer are shown in Figure 1(d,e). This sensing layer has a typical fiber network-like structure. The diameter of a single yarn is about 10 μm with ample free spaces between the microfibers. Conductive particles (CNTs) can be observed in the form of thin coatings and stuck randomly onto the pristine surface (tissue paper). These CNT particles covered the fibers without any significant crack. Figure S2 shows the cross-section SEM image, which is favorable to the sensor’s operation and stability.

## 3. Results and discussion

**Figure 2.**
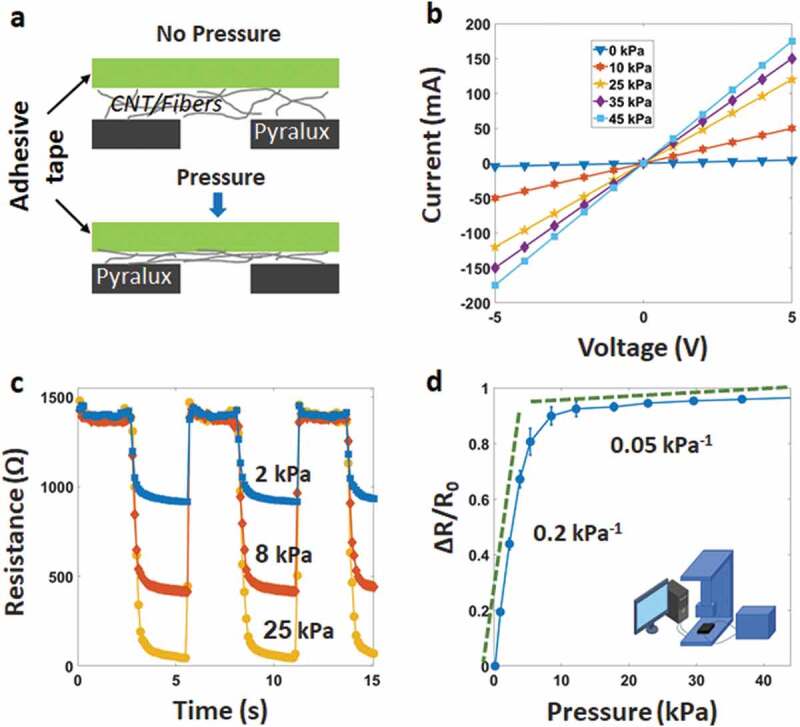
(a) Working principle of the sensor, (b) I–V curves at the different pressures from −5 V to 5 V, (c) Pressure–resistance relationship at the different pressures, (d) Relative resistive change as a function of pressure, showing the sensitivity of the sensor

Figure 2(a) shows the working principle attributed to the change of the pressure-dependent contact resistance between the sensing layer and the interdigitated electrodes. These layers are assembled face to face under unloading conditions. Some conductive fibers protrude and connect the electrodes, while some others are not. Under loading conditions, the resistance variation relies mainly on the increase in the contact area between the tissue-CNT fibers [44,45]. This leads to a decrease in resistance. After removing the pressure, the contacted numbers of the protruded fibers decrease, resulting in recovering of the sensor resistance.

To analyze the fabricated sensor’s electrical characteristics, we used a universal testing machine (UTM), consisting of a source meter and a controlling computer, as described in Figure S3. The pressure sensor was placed above the sole. Two electrodes of the sample directly connected two grips of the UTM. Resistance and pressure were continuously measured and recorded average values during the experimental. Figure 2(b) shows a set of the current-voltage (I–V) graphical curves under different static pressures at 0, 10, 25, 35, and 45 kPa. As seen, the applied voltage from – 5 to 5 V demonstrates good linearity, indicative of an ohmic behavior.

The resistance of the sensor decreases with increasing pressure (Figure 2(c)). Figure 2(d) shows the sensitivity (*S* = *δ*(Δ*R*/*R*_0_)/*δP*) at the different levels of the loading pressure, where R_0_, R, and P present the initial resistance, the resistance with applied pressure, and the pressure change, respectively. Ten samples were tested, and the average value was recorded. In Figure 2(d), error bars represent the standard deviation of data. The sensitivity can be found to be ~0.2 kPa^−1^ in the low-pressure range (<6 kPa). However, it drops to about 0.05 kPa^−1^ at the pressure over 8 kPa, due to the contact area becomes saturated under high compression.

**Figure 3.**
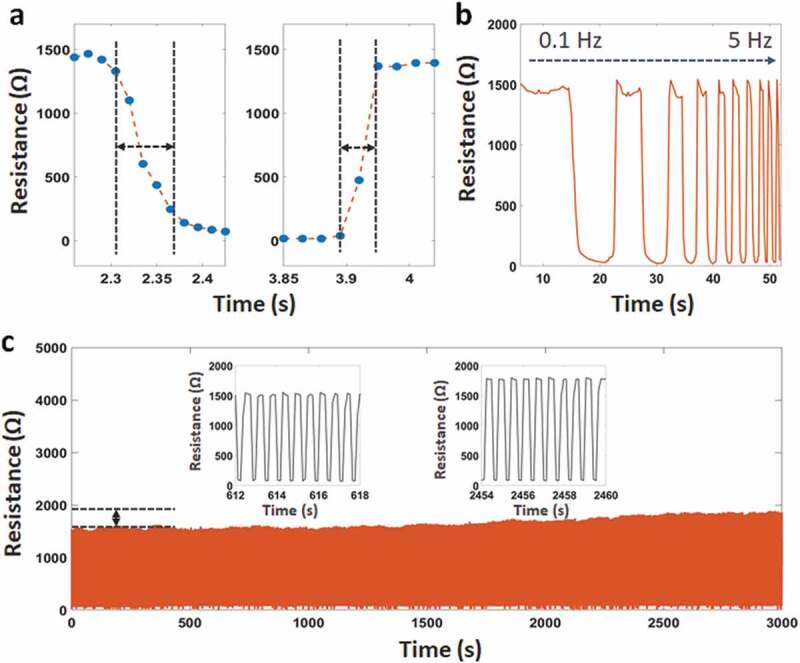
(a) Response-relaxation time, (b) Resistance change at the different frequencies from 0.1 Hz to 5 Hz, (c) Durability of the sensor after 5,000 loading/unloading cycles

As described in Figure 3(a), the response and relaxation times can be estimated as short as 70 and 50 ms, respectively. Delay time is caused by the viscoelastic nature of the fibers and the connectivity between CNT coatings under pressure. Here, the protruded structures on the sensing layer and the electrodes enable their surfaces to produce the elastic deformation and minimize the viscoelasticity. We also investigated the dynamic performance of the sensor in Figure 3(b). It is clear that the sensor has a stable response under a wide mechanical frequency range from 0.1 to 5 Hz. Besides, Figure S4 shows the curve of the resistance change in the bending test at different bending radius (22.5 mm, 15 mm, and 10 mm), as well as the resistance change before and after bending deformation. The resistance results at each pressure value describes no appreciable degradation compared with the as-prepared sensor. The sensor also demonstrates a small hysteresis with a pressure between 0 ~ 45 kPa (Figure S5). The above properties will ensure the rapid electrical property of the sensor in practical applications.

Figure 3(c) describes the stable electrical functionality and mechanical integrity during loading/releasing cycles. We observed that the dynamic durability of the sensor is over 5,000 cycles. There is a small difference at the maximum resistance ~7% after 2,000 cycles and ~10% after 4,000 cycles. The reason for this behavior is the permanent deformation in the structure under pressure leading to a small change in the maximum resistance. We predict that CNT particles have fallen out of the fibers during working, resulting in a maximum resistance increasing gradually in the following cycles. To overcome this issue, we suggest using CNT or Ag pastes to increase the connection between the conductive particles and the fibers. However, this solution will increase the cost and require more experiments.

**Figure 4.**
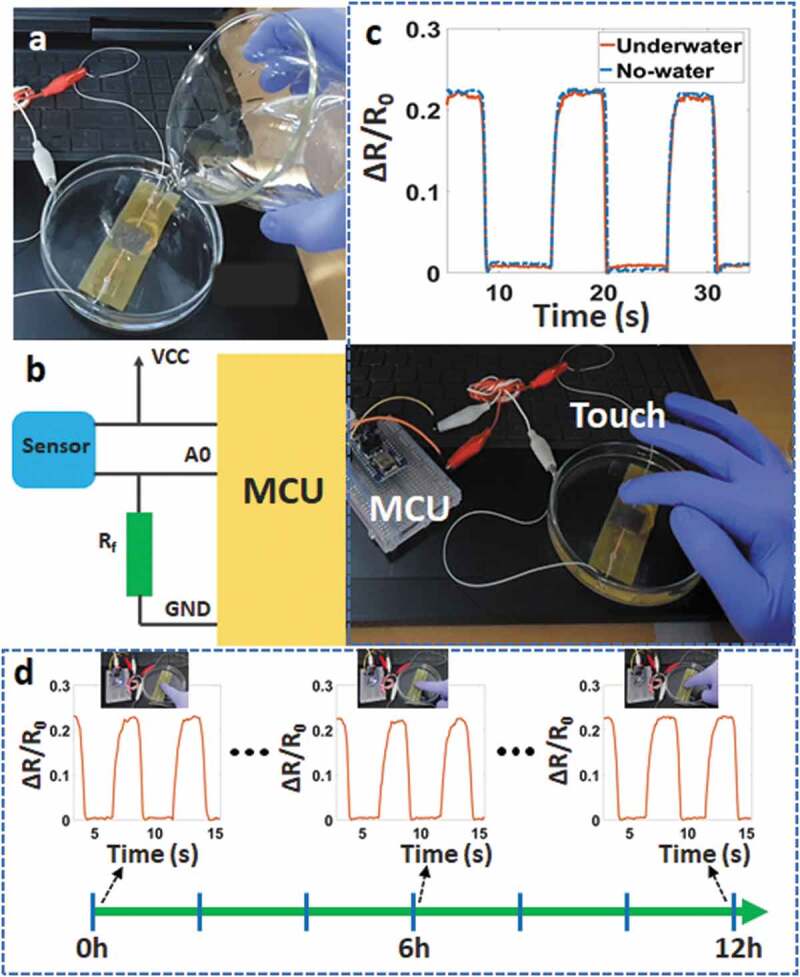
(a) The sensor underwater, (b) Schematic of the testing circuit, (c) Signal change with applied pressure underwater and no-water, (d) Touch signal of the sensor when immersed in the water

One highlight of this sensor is the ability to waterproof by the polyimide and the adhesive layers. The soldered contacts, LED, IC, and other components of the circuit are protected by one coating layer (adhesive tape). In other words, our sensor can work underwater. Figure 4(a-c) and Video S1 demonstrate the signal change under applied pressure, while all sensor parts are immersed in the water (not the deionized water). In the experiment, the testing circuit is a voltage divider circuit with an nRF52 module as a microcontroller and a reference resistor (1 kΩ). There is almost no difference in the observed signal with the case of the water-absence. In addition, the lifetime underwater of the sensor is also characterized in Figure 4(d). We observed that the touch signal is not changed at the different testing times (at 0 hours, 6 hours, and 12 hours). This will ensure the ability of the sensor to work underwater for a long time.

Table 1 summarizes some flexible sensors in the previous studies – based on hand-drawing/paper apporach, consisting of materials, principle, sensitivity, working range, response time, and waterproof capabilities [37,40–42,46–50]. Most of the sensors were fabricated from copy paper and pencil. Some of them combined the silver conductive pen and tissue/GO-papers. It is clear that our sensors are thin, even when the sensitivity is higher than those of other studies and close to the capacitive pressure sensors. Especially, our sensors are hand-drawing and waterproof. This capability will fit many wearable applications, such as devices-in-rain, scuba diving clothes, or diving robotics.

**Table 1.** Summarizes flexible sensors – based on hand-drawing/papers

Materials/Components	Type of sensor	Principle	Range	Sensitivity	Thickness	Response/Relaxation time	Hand drawing	Waterproof	Ref.
Cellulose paper, PDMS, Graphite (Pencil), ITO NPs	Bending	Resistive	-	-	< 140 µm	-	Yes	No	[37]
Cellulose paper, Graphite (Pencil)	Temperature, Biochemical,Electrophysiological	Resistive	-	-	< 110 µm	-	Yes	No	[40]
Copying tissue paper, Graphite (Pencil), GO & fiber foam	Pressure	Capacitive	0–50 kPa	~ 0.14 kPa^−1^	~ 110 µm	180/120 ms	Yes	No	[41]
Cellulose paper, Graphite (Pencils), Silver ink, Nickel ink, IGZO semiconductor	Pressure, Op-amp	Resistive	0–1.2 kPa	~ 0.05 kPa^−1^	-	-	Yes	No	[42]
Cellulose paper, Conductive brush pen	Strain	Resistive	-	< 0.001 kPa^−1^	~ 150 µm	-	Yes	No	[46]
Post-it paper, Silver ink pen, Graphite (Pencil), Aluminum foil, Microfiber Wipe, Sponge	Pressure/Temperature/Humidity/PH	Resistive/Capacitive	-	0.16 kPa^−1^	-	-	Yes	No	[47]
Airlaid paper, Carbon black, Copper tape, Semipermeable film	Pressure	Resistive	0–30 kPa	> 7.12 kPa^−1^	-	200 ms	No	Yes	[48]
Graphite (Pencil), Polyester substrate	Electrodes	-	-	-	-	-	Yes	No	[49]
Cellulose paper, Graphite (Pencil)	UV, Pressure, Chemical, Glucose	Resistive	-	~ 0.009 kPa^−1^	-	-	Yes	No	[50]
Flexible film (pyralux), Permanent marker pen, Wet-tissues, CNTs	Pressure, Resistor	Resistive	0–45 kPa	0.2 kPa^−1^ (0–6 kPa), 0.05 kPa^−1^ (> 8 kPa)	~ 260 µm	50/70 ms	Yes	Yes	Ours

**Figure 5.**
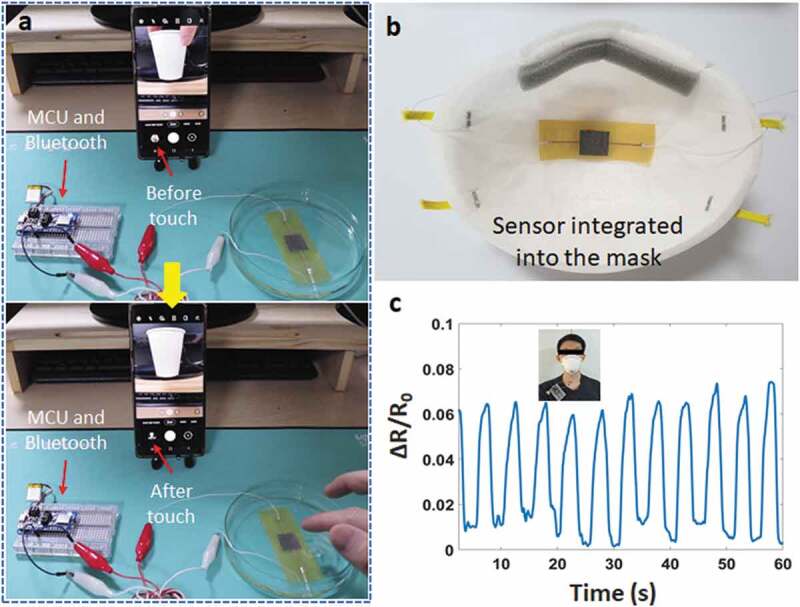
(a) Taking a photo on phone when dipping the sensor underwater, (b) Sensor integrated into the mask, (c) Breathing signal

To demonstrate the potential applications, our sensor was tested to take a photo on phone when it was immersed in the water (Figure 5(a)). Signal processing system is an integrated circuit (Figure S6), consisting of an nRF52 module (analog/digital converter – ADC, microcontroller unit – MCU, Bluetooth) and a lipo-battery (3.7 V). Analog signals were sampled/digitized and converted into digital signals. The resolution was set up between 0 and 3 V into digital values between 0 and 1023 (3/1023 ~ 0.003 V or 3 mV per unit). The input signal was read every 25 ms. So, the reading rate is 40 times per second. The testing process can be observed in Video S2. Our sensor showed a high and stable performance, even with a slight touch.

Breathing rate monitoring is an example of this sensor in the healthcare field. Figure 5(b) describes the pressure sensor embedded in a smart mask and the signal amplitude shows the mouth breathing rate. The high sensitivity makes it possible to detect small pressure when breathing. We used an amplifier module to increase the breathing signal variation, and a good result can be seen in Figure 5(c).

**Figure 6.**
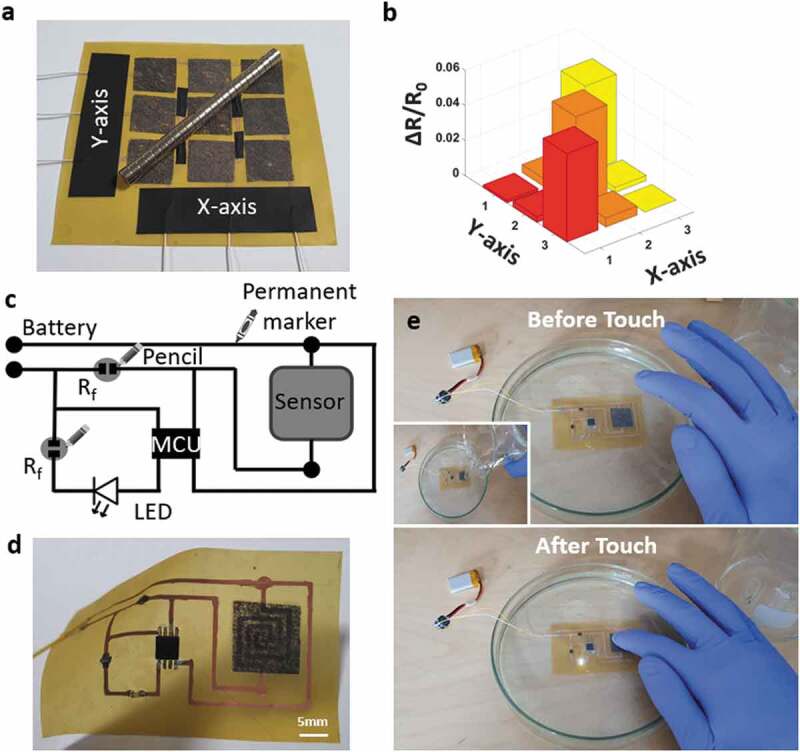
(a) Structure of the matrix sensing layer, (b) Signal change of the matrix layer when applied an object, (c) Schematic of the hand-drawing circuit, (d) Real picture of the hand-drawing circuit, (e) Hand-drawing circuit when working underwater

For *e*-skin applications, this sensor is a highlight with the small thickness and the scalability. Figure 6(a) shows a sensor array that can recognize the stimulus as well as reflecting the position of the stimulus. The individual sensors in this array were connected as shown in Figure S7 with four jump-connections. The weight of the object is about 15 g. Using a signal scanning method to detect the position, we can easily collect the resistance change of each sensor node (Figure 6(b)).

On the other side, our method also desmonstrates the possibility of a fully integrated circuit that is created by hand-drawing and can work underwater. The circuit diagram can be seen in Figure 6(c-d). Herein, an Attiny85 works as a simple microcontroller unit. Two reference resistors (R_f_) are 1–2 kΩ and drawn directly on the pyralux film by a pencil. One resistor used to protect the LED, and another resistor used to detect the change in the voltage divider circuit. As shown in Figure 6(e), the integrated circuit is programmed to turn on an LED when touching the sensor. Video S3 demonstrated the practical operation of this integrated circuit underwater. Two existing hard-components are the MCU and LED. However, this issue also opens a research direction for an all-hand-drawing-circuit in the future.

**Figure 7.**
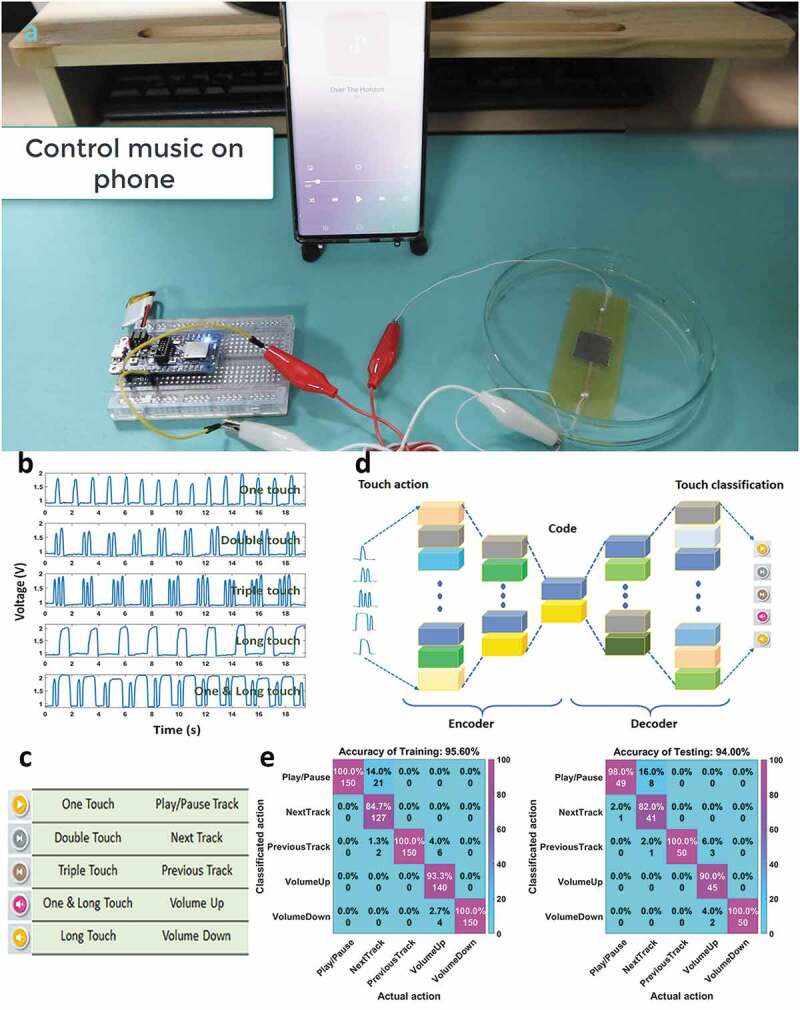
(a) The experimental diagram to control music on phone, (b) Signal of each touch action, (c) Controlling commands corresponding to touch actions, (d) Structure of the autoencoder model, (e) Confusion matrices of the training data and testing data

As mentioned above, the final experiment is controlling music mode on a phone with different touch actions when the sensor is dipped underwater. The experimental diagram can be seen in Figure 7(a). The sound of the tracks can be played directly on the phone or via Bluetooth earphones. It demonstrates that the wireless connection between the sensor and the phone does not affect the wireless connection between the phone and other devices. Signal patterns for five actions, including one-touch, double-touch, triple-touch, one & long-touch, long touch, are shown in Figure 7(b). Corresponding to five actions, there are five commands to control music mode on a phone (Figure 7(c)), including play/pause track, next track, previous track, volume up, and volume down. The signal from the sensor is recorded with 200 samples for each action, where 150 samples used for training (75%), and 50 samples used for testing (25%). Total is 1,000 samples of all actions.

As described in Figure 7(d), the application is built with a machine learning algorithm (autoencoders) to classify those actions. Accordingly, the depth of the encoders is three hidden layers, which are 100, 50, and 10 dimensions. A softmax layer with the cross-entropy is used as the activation function for the classification. After the training process in the autoencoders model, a high classification accuracy of 94% is achieved as depicted in the confusion matrix of Figure 7(e). This value indicates that there is a good agreement between the touch actions and the classified actions. While the triple-touch actions, long-touch actions are easy to classify (100%), the double-touch actions (82%) can be confused with the one-touch actions (98%). Besides, the one & long touch actions have an accuracy of 90%. Detailed Video S4 in the Supplementary data can be found in supporting materials. This demonstration provides a great potential for control based on motion classification of the signal patterns through machine learning and our hand-drawing sensor.

## 4. Conclusions

In summary, we have presented a waterproof, thin, and high-performance pressure sensor via the hand-drawing process. The basic structure of the sensor is as follows: (i) flexible pyralux film with 1 oz copper and polyimide, (ii) sensing layer with wet-tissues-coated by CNTs, and (iii) thin adhesive layers. The obtained sensor is easy to create (hand-drawing) without any designing software. The small thickness (~0.26 mm) and high-performance at the sensitivity of 0.2 kPa^−1^ (<6 kPa) help to increase the applicability of the sensor in many areas, such as human-machine controlling, healthcare, or *e*-skins. Multifunctional applications were demonstrated in the experiments of taking a photo on the phone, monitoring breathing rate when using a mask, and sensing array when touch or multi-touch. The biggest advantage of this sensor is the ability to work underwater that expands the limit of the typical hand-drawing sensors. Finally, by applied machine learning, the touch actions underwater achieved high classification accuracy (94%), opening an expectation of a complete system for diving applications. Looking forward, the combination of sensors and machine learning will become a universal platform for various recognition tasks of complex motions.

## Supplementary Material

Supplemental Material

Supplemental Material

Supplemental Material

Supplemental Material
